# Efficacy of the Ascure Smoking Cessation Program: Retrospective Study

**DOI:** 10.2196/17270

**Published:** 2020-05-14

**Authors:** Ayaka Kato, Tomoyuki Tanigawa, Kohta Satake, Akihiro Nomura

**Affiliations:** 1 CureApp Institute Karuizawa Japan; 2 Department of Life Sciences Graduate School of Arts and Sciences The University of Tokyo Tokyo Japan; 3 CureApp Inc Tokyo Japan; 4 Japanese Red Cross Medical Center Tokyo Japan; 5 Innovative Clinical Research Center Kanazawa University Kanazawa, Ishikawa Japan; 6 Department of Cardiovascular Medicine Kanazawa University Graduate School of Medical Sciences Kanazawa Japan

**Keywords:** smoking cessation, nicotine dependence, telecare, telemedicine, mHealth, digital therapeutics, mobile phone, smoking cessation program, online counseling

## Abstract

**Background:**

Smoking cessation helps extend a healthy life span and reduces medical expenses. However, the standard 12-week smoking cessation program in Japan has several notable problems. First, only 30% of participants complete this program. Second, participants may choose not to participate unless they have a strong motivation to quit smoking, such as health problems. Third, the program does not provide enough support during the period between clinical visits and after 12 weeks.

**Objective:**

This study examined the efficacy of the 24-week ascure program to address the problems of accessibility and continuous support. The program combines online mentoring, over-the-counter pharmacotherapy, and a smartphone app.

**Methods:**

Using a retrospective study design, we investigated data for 177 adult smokers who were enrolled in the ascure smoking cessation program between August 2017 and August 2018. The primary outcomes were continuous abstinence rates (CARs) during weeks 9-12 and weeks 21-24. To confirm smoking status, we performed salivary cotinine testing at weeks 12 and 24. We also evaluated the program adherence rate. Finally, we performed exploratory analysis to determine the factors associated with continuous abstinence at weeks 21-24 to provide insights for assisting with long-term continuous abstinence.

**Results:**

The CARs of all participants for weeks 9-12 and weeks 21-24 were 48.6% (95% CI 41.2-56.0) and 47.5% (95% CI 40.0-54.8), respectively. Program adherence rates were relatively high throughout (72% at week 12 and 60% at week 24). In the analysis of the factors related to the CAR at weeks 21-24, the number of entries in the app’s digital diary and number of educational videos watched during the first 12 weeks were significant factors.

**Conclusions:**

The ascure program achieved favorable CARs, and participants showed high adherence. Proactive usage of the smartphone app may help contribute to smoking cessation success in the long-term.

## Introduction

### Background

Smoking is a preventable cause of many health problems, such as cardiovascular diseases, respiratory diseases, and malignant tumors [1,2]. There are more than 20 million smokers in Japan, and deaths from smoking-related diseases are estimated to exceed 12,900 annually [3]. Smoking cessation can greatly contribute to extending a healthy life span and the reduction of medical expenses [3,4]. In Japan, smoking cessation is mainly encouraged through outpatient services. Following a diagnosis of nicotine dependence via interviews and questionnaires at their first visit, patients are eligible for a 12-week standard smoking cessation treatment program that includes 5 clinic visits and an initial screening interview to check exhaled carbon monoxide [5]. In many cases, respiratory physicians and nurses administer the cessation treatment program, which includes the following elements: evaluating the severity of nicotine dependence, measuring the exhaled carbon monoxide concentration, prescribing the appropriate medicine (mostly varenicline or a nicotine patch), and a consultation for quitting smoking, with advice based on behavioral therapy.

This standard smoking cessation program, however, has 3 major problems. First, only about 30% of participants who start the program complete it, often because of the burden of the clinical visits [5]. Second, there is a gap between the number of people who want to quit smoking and those who go to a hospital. The estimated ratio of smokers who participated in the program is only approximately <5% [5,6], although 29.8% of smokers are willing to quit smoking [7]. Participants who choose to join the program might only do so because they have serious health problems or a strong motivation to quit smoking; for example, 38% of outpatient program participants had coexisting diseases [5]. According to pooled analysis from 8 cohort studies in Japan [8], to lower the risk of morbidity from smoking-related cancer to the same level as that of a lifetime non-smoker, male smokers needed over 21 years, and female smokers needed 11 years of abstinence. Therefore, early intervention is important, especially encouraging quitting in young people who might not yet have coexisting diseases or who might be motivated to quit. Third, the continuous abstinence rate (CAR) drops significantly after completing the 12-week program and pharmacological therapy [9]. Given that the decline in CAR plateaus at around week 24, finding a way to assist patients in continuing abstinence beyond 12 weeks could help ensure long-term smoking cessation success [9].

As one way to address the shortcomings of the 12-week program, the “ascure” smoking cessation program was created by CureApp Inc. Its features address some of the concerns related to the standard 12-week program: It is conducted remotely; uses a smartphone app, which is familiar to young people, to provide follow-up assistance; and supports participants for 24 weeks. It also provides professional online mentoring and offers nicotine replacement therapy (over-the-counter medical patches) delivered to the patient’s home. The ascure smartphone app was developed based on research with the CureApp Smoking Cessation app [10-12]. App users have access to tailored guidance in a timely fashion through daily use. The ascure program also offers 6-8 online counseling sessions conducted with experienced nurses and pharmacists.

Smoking cessation programs based on smartphone apps are rapidly increasing globally [13,14]. Despite their popularity, there is limited research on which aspect of the apps predicts smoking cessation. A study on an app employing a behavior change model called acceptance and commitment therapy (ACT) and investigating the associations between a user's smoking cessation and their usage of the app features reported that 2 ACT-specific practices and viewing the cessation plan were the strongest predictors of smoking cessation [15]. Another study analyzed a smoking cessation app designed to deliver the essential features of the United States Clinical Practice Guideline [16]. The authors reported that the number of weeks of active app use (>0 interactions/week) was an important predictor of successful smoking cessation among those using the app. However, no systematic analysis has been done that considers the predictive factors of smoking cessation, especially of app features, when using a combination of online mentoring, over-the-counter medication, and a smartphone app among Japanese smokers.

### Objectives

This study retrospectively examined the clinical efficacy of the ascure smoking cessation program and its impact on continuous smoking cessation. We also used exploratory analysis to determine the factors associated with continuous abstinence at weeks 21-24 to gain insights for supporting long-term continuous abstinence.

## Methods

### Study Design

This was a retrospective study evaluating the efficacy of the ascure smoking cessation program. We conducted this study in compliance with the Declaration of Helsinki, and all other applicable laws and guidelines in Japan. All study procedures were reviewed and approved by the Kanazawa University Institutional Review Board (2019-023 (3058)).

### Participants

We assessed 177 adult smokers participating in the ascure smoking cessation program in Japan from August 2017 to August 2018. All participants had their own iOS or Android smartphones, desired to quit smoking, and were members of one of 16 corporate health insurance societies. Participants were recruited mostly via self-selection. Some became aware of this program through individual recommendations from professionals (public health nurses) at medical checkups. Advertisements by email, post, or leaflets distributed at hospitals were used for recruitment. We included participants who smoked >10 cigarettes per day and who agreed to the study’s privacy policy by providing written informed consent for data to be used in our analysis. We excluded participants who had difficulty using their smartphones according to the instructions or who were diagnosed with a mental illness.

### Outcomes

The primary outcomes of this study were participants’ CARs during weeks 9-12 and weeks 21-24. During the online video sessions at weeks 12 and 24, mentors asked participants whether they had smoked during the month prior to the current session and asked them to perform salivary cotinine testing using iScreen (Cotinine Oral Fluid Screening Device, Abbott Diagnostics Medical Co, Ltd, Tokyo, Japan). The results of the test were confirmed visually via video. This allowed supplementation of the self-reported continuous abstinence via salivary cotinine testing. We also assessed adherence to the program based on how many weeks participants took part in the online sessions (weeks 1, 2, 4, 8, 12, and 24). Furthermore, we used exploratory analysis to determine the variable factors associated with smoking cessation success at week 24 (demographic data, smoking history, and app usage) to provide potential insights for maintaining long-term continuous abstinence.

### Ascure Smoking Cessation App

The ascure smoking cessation program is a 24-week completely remote, online program including 6 online sessions with the exclusive ascure smoking cession smartphone app. The app was developed by CureApp Inc (Tokyo, Japan). As noted earlier, the app’s contents were developed based on the findings of clinical studies using the CureApp Smoking Cessation app for patients diagnosed with nicotine dependence (Multimedia Appendix 1). The ascure app is compatible with both iOS and Android smartphones and meets the software inspection criteria and security requirements of the Apple App Store and Google Play. CureApp Inc provided participants with the app activation codes to begin use. The participants downloaded the app, activated the app using the codes provided, and entered their personal demographic information including age, gender, years of smoking, and estimated number of cigarettes smoked per day. Their information was securely stored on a cloud system and utilized to support the smoking cessation of each participant in their personalized counseling.

**Figure 1:**
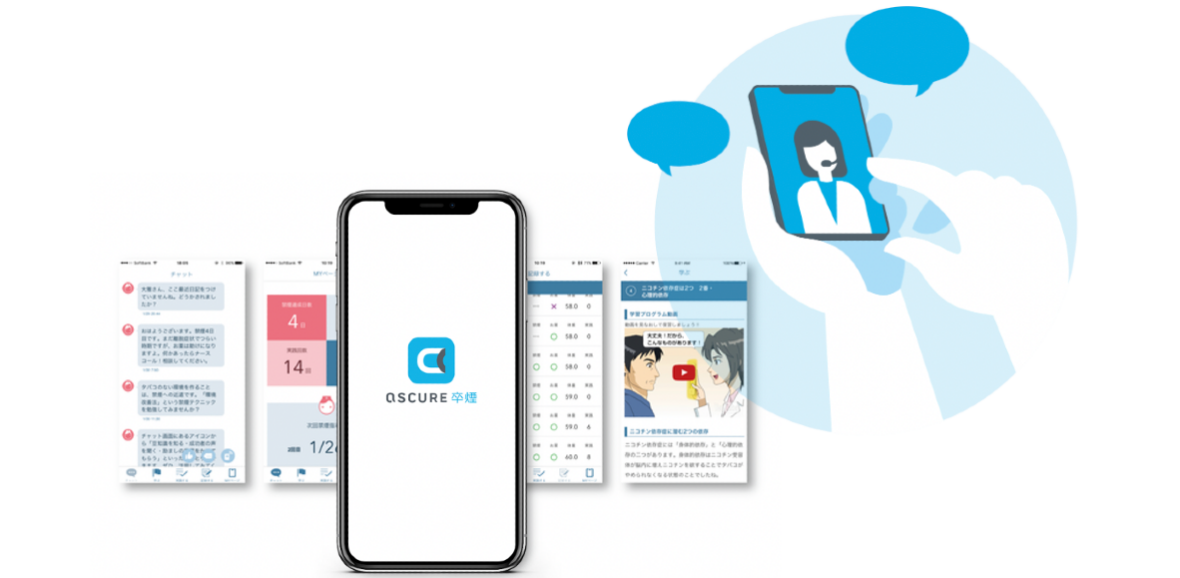
Scheme of the ascure smoking cessation program.

The app (see Figure 1) comprises 4 steps to maximize the therapeutic effect of the medications and online mentoring sessions for smoking cessation: learning, exercise, keeping a record, and rescue.

#### Learning: Educational Video Tutorials to Help Users Quit Smoking

Participants were encouraged to watch 1-3–minute video tutorials on nicotine dependence that provided useful tips on smoking cessation based on behavioral therapy. A total of 24 video tutorials were provided during the 24-week smoking cessation program.

#### Exercise: A Personalized To-Do List to Change Habits

After being exposed to the behavioral therapy through the tutorials, participants could make a personalized to-do list for quitting smoking. For example, they could list alternative behaviors such as brushing their teeth or stretching when they craved a cigarette. When participants carried out the registered alternative behavior, they could record it in the digital diary provided in the app.

#### Keeping a Record: Digital Diary of Smoking Cessation

The app instructed participants to keep a digital diary on their smoking cessation status, physical condition, medication use, and any adverse events, either by selecting from prepopulated options or by writing entries in free form.

#### Rescue: Interactive Chat Sessions

Whenever participants experienced cravings or withdrawal symptoms, they could tap the “call” button to send a message to a personalized chatbot. The chatbot immediately replied and provided personalized advice on how to deal with the symptoms. The chatbot also provided encouraging messages on smoking cessation to app users to remind the participants of their motivation to quit smoking.

### Online Mentoring

Throughout the 24-week program, 6-8 interactive online sessions with experienced mentors (normally professional nurses or pharmacists) were provided. Participants met with mentors via a web-based counseling system at each planned visit (weeks 1, 2, 4, 8, and 12, as in the standard smoking cessation outpatient program, with an additional session in week 24). During the online sessions, the mentors asked participants about their smoking status and any difficulties they encountered. Participants were also provided with guidance on overcoming psychological dependence. Each online mentor in the ascure program had more than 3 years of experience as a nurse, public health nurse, dietitian, or pharmacist. All instructors joined the Japan Society of Tobacco Control or The Japanese Association of Smoking Control Science and were qualified by either society before their initial mentoring session. In-house training was conducted for 1 month to learn the system for smoking cessation.

### Medication

As part of the program, participants received over-the-counter nicotine patches or nicotine gum as a smoking cessation aid, which was delivered via parcel from a pharmacy (ascure store [former Nihonbashi Smart Clinic], Tokyo, Japan) to their homes. This nicotine replacement treatment was typically used until week 8.

### Data Collection

We collected basic information on each participant via the app, including their age, gender, years of smoking, number of cigarettes smoked per day, and screening of tobacco/nicotine dependence using methods such as the Tobacco Dependence Screener (TDS) score [17], Fagerström Test for Nicotine Dependence score [18], and Kano Test for Social Nicotine Dependence (KTSND) score [19], the metrics of which focus on their psychological dependence. We also gathered app usage statistics, such as the number of days that participants updated their diaries, number of “call” button presses, and number of educational videos viewed.

### Statistical Analysis

Continuous variables are presented as mean (SD) or median (interquartile range [IQR]), depending on their distribution. Categorical variables are presented as the number (percentage). For the exploratory factor analysis of the predictors of CAR at week 24, we conducted multivariable logistic regression analysis, selecting from among the standardized variables (demographic data, smoking history, and app usage evaluated between weeks 0 and 12), while adjusting for age, gender, and TDS score. In multiple testing, the Bonferroni method was used to adjust the *P* values. *P* values <3.0 x 10^-3^ (0.05 divided by 15) were considered to be significant. There were 36 missing values for the TDS score and 40 missing values each for the Fagerström Test for Nicotine Dependence and KTSND. To address these missing data in the explanatory variables, we used a multiple imputation method. We used the Pearson Chi-square test for subpopulation analysis. All calculations and analyses were performed using *R* software version 3.5.2 (*R* Foundation for Statistical Computing, Vienna, Austria); packages used were ‘data.table,’ ‘stats,’ and ‘mice’.

## Results

### Baseline Characteristics

Table 1 shows the baseline characteristics of the 177 participants.

**Table 1:** Baseline characteristics of the 177 participants.

Characteristics	Values	Range
Age (years), mean (SD)	44.6 (9.7)	26-63
Female gender, n (%)	65 (36.7)	N/A
Brinkman index, mean (SD)	362 (228)	20-1260
Cigarettes per day, mean (SD)	16.2 (6.4)	2-40
Years of smoking, mean (SD)	22.0 (9.8)	2-42
Number of smoking cessation attempts before the trial, median (IQR^a^)	1 (3)	0-11
TDS^b^ score, median (IQR)	8 (3)	0-10

^a^IQR: interquartile range.

^a^TDS: Tobacco Dependence Screener.

### Efficacy of the Ascure Program

Program adherence was 71.8% (127/177) at week 12 and 59.9% (106/177) at week 24. Within the first 4 weeks of the program, 23 people did not book the subsequent online mentoring sessions, and 11 and 16 people, respectively, lost contact with their mentors during the week 4 and week 8 sessions. The biochemically validated CAR during weeks 9-12 was 48.6% (86/177; 95% CI 41.2-56.0; Table 2). At weeks 9-12, 86 people had successfully quit smoking, while 50 people could not be evaluated because they dropped out of the program. The biochemically validated CAR during weeks 21-24 was 47.4% (84/177; 95% CI 40.0-54.8). We confirmed continuous adherence for 84 of the 106 individuals who completed the full 24-week program. Of these, 75 people also succeeded in 1 month of smoking cessation during weeks 9-12, and 9 people who had been counted as unsuccessful at week 12 became successful at week 24. No participants reported adverse events, including app-related events, during the program other than skin rashes and nausea caused by nicotine patches.

**Table 2:** Efficacy of the ascure program.

Measurement	Efficacy rate, n (%)	95% CI
CAR^a^ at weeks 9-12	86 (48.6)	41.2-56.0
CAR at weeks 21-24	84 (47.4)	40.0-54.8

^a^CAR: continuous abstinence rate.

### Predictors of CAR at Weeks 21-24

Next, we evaluated the predictors of CAR during weeks 21-24 (Table 3). The candidate factors were the demographic variables, smoking history, and app usage. The learning comportment of the app was quantified by the number of educational video tutorials watched. A personalized to-do list to change habits included the number of times environmental triggers were avoided, number of behavioral patterns changed, number of alternative behaviors, and number of instances of assertiveness training. The number of diary entries and reports of poor physical condition reflected the use of the recording function of this app. The use of the interactive chat was measured by the number of “like” button presses and nurse “call” button presses. The multivariable analyses revealed that the number of days participants wrote in their digital diaries and number of educational video tutorials watched during the first 12 weeks were significantly associated with CAR during weeks 21-24.

Moreover, we calculated the CARs at weeks 21-24 separately by subgroups of the number of days that participants wrote in their diaries. Participants who wrote diary entries more than the median number (43 times; range, 0-77 times) during the first half of the program achieved higher CARs (59/88, 67%) than those who did not (25/89, 28%; χ^1^_177_ = 25.39, *P*<.001) when counting dropout as a failure. Even within the group of 106 people who completed all 24 weeks, the subgroup who wrote more diary entries than the median number (62.5 times) had a higher success rate (47/53, 88.7%) compared with those who did not (37/53, 69.8%; χ^1^_106_ = 4.65, *P*=.03).

Participants who watched educational videos more than the median number (6 times; range, 0-24 times) also had significantly higher CARs (55/86, 64.0% vs. 29/91, 31.8%) when counting dropout as a failure (χ^1^_177_ = 16.99, *P*<.001). However, there was no significant difference between success or failure among people who completed all 24 weeks (χ^1^_177_ = 2.19, *P*=.14).

**Table 3:** Results of multivariable logistic regression analysis to determine potential predictors of smoking cessation success at week 24.

Variable		Odds ratio	95% CI	*P* value	AIC^a^
**Demographic data**					
	Age	1.577	1.159-2.174	.004	239
	Sex (male: 0, female: 1)	0.552	0.289-1.039	.068	239
**Smoking history^b^**					
	Years of smoking	1.405	0.840-2.409	.197	239
	Number of cigarettes/day	1.011	0.743-1.375	.944	241
	Number of smoking cessation attempts before the trial	0.838	0.604-1.140	.268	240
	TDS^c^	0.752	0.537-1.036	.087	238
	FTND^d^	0.791	0.537-1.081	.146	239
	KTSND^e^	0.747	0.523-1.043	.095	238
**Application usage^f^**					
	Educational video tutorials watched	2.178	1.550-3.126	<.001	219
	Times environmental triggers were avoided	2.441	1.367-5.394	.011	229
	Times behavioral patterns were changed	3.184	1.550-9.029	.009	226
	Alternative behaviors	2.514	1.404-5.922	.010	228
	Instances of assertiveness training	1.560	1.017-2.896	.089	236
	Diary entries	2.661	1.866-3.894	<.001	208
	Reports of poor physical condition	1.172	0.860-1.630	.320	239
	“Like” button presses	1.638	1.108-2.673	.026	237
	Nurse call button presses	1.510	1.059-2.366	.040	235

^a^AIC: Akaike information criterion.

^b^Adjusted by age and gender.

^c^TDS: Tobacco Dependence Screener.

^d^FTND: Fagerström Test for Nicotine Dependence.

^e^KTSND: Kano Test for Social Nicotine Dependence.

^f^Adjusted by age, gender, and TDS.

### Exploratory Analyses

As a reference, we reviewed the efficacy of the standard smoking cessation program. There are several differences in the baseline characteristics between the ascure program and the standard program. Compared with the national survey (Table 4), the ascure program participants were relatively young, more often female, and had low dependence, as represented by the Brinkman index.

While only 29.8% (390/1308) of smoking cessation outpatients completed the standard 12-week treatment, ascure participants had a higher program adherence rate of 71.8% (127/177) at week 12 and 59.9% (106/177) even at week 24.

Although the efficacy of the 2 programs cannot be compared directly, both programs measured efficacy with a 1-month CAR at weeks 9-12. In the case of the outpatient service, the self-reported 1-month continuous abstinence was confirmed by exhaled carbon monoxide concentration (<8 ppm) at the fifth clinical visit.

Among the participants continuing the ascure program until week 12, the CAR at week 12 was 67.7% (86/127; 95% CI 59.4-76.0), whereas the CAR among individuals who completed the 12-week outpatient program was 82.1% (320/390) [5]. However, in contrast to the 12-week outpatient program, the ascure program assisted participants for up to 24 weeks through the smartphone app. The CAR at weeks 21-24 among people who completed the ascure program was 79.2% (84/106; 95% CI 71.4-87.1). Importantly, when counting dropouts as failures, the CAR of all ascure participants at weeks 9-12 (86/177, 48.6%) was twice that of outpatients in the standardized smoking cessation program (320/1308, 24.5%) [5].

**Table 4:** Baseline characteristics of the participants.

Characteristic	Standard program, n=1308	ascure program, n=177
Age (years), mean (SD)	49.0 (14.5)	44.6 (9.7)
Female sex, n (%)	400 (30.6)	65 (36.7)
Brinkman index, mean (SD)	634.1 (448.3)	362 (228)
Cigarettes per day, mean (SD)	22.8 (10.2)	16.2 (6.4)
Years of smoking, mean (SD)	27.5 (13.4)	22.0 (9.8)
TDS^a^ score, median (IQR^b^)	8 (N/A^c^)	8 (3)

^a^TDS, Tobacco Dependence Screener.

^b^IQR: interquartile range.

^c^IQR not reported in the publication.

## Discussion

### Principal Findings

This study evaluated the efficacy of the ascure smoking cessation program, which is characterized by online mentoring, a smartphone app, over-the-counter medications, and an extended follow-up period (24 weeks). First, we found that the CAR was 48.6% during weeks 9-12 and 47.5% during weeks 21-24. We also observed high adherence rates: 72% at week 12 and 60% at week 24. Unlike previous reports [9], CARs did not decrease significantly between weeks 12 and 24. Second, using multivariable logistic regression analysis, we found that the number of days of digital diary entries and number of educational videos watched during the first 12 weeks were significantly associated with the CAR at weeks 21-24. The number of days of digital diary entries was significantly different between people who succeeded or failed in 1-month continuous abstinence at week 24 even when dropouts were excluded.

### Comparison With Prior Work

This study has 3 important findings. First, the ascure smoking cessation program achieved moderately favorable CARs in comparison with the current standard Japanese outpatient program, mainly due to the adherence rate. While only about 30% of participants completed the outpatient program [5], the completely remote ascure program saw a higher adherence rate, even at week 24. Participants in the ascure program achieved reasonable CARs without face-to-face advice from medical doctors; without taking varenicline, a prescription drug that blocks the pleasant effects of nicotine; and without the need to visit smoking cessation clinics. This program is intended to provide an alternative to the standard program for those who cannot access the standard program, including busy professionals. This program could be convenient for people who do not visit hospitals frequently, who do use a smartphone frequently, and who are familiar with web-conferencing. On the other hand, the outpatient service is a better solution for those who have other serious health problems or have experienced withdrawal symptoms. The differentiating aspects of ascure from other app-based smoking cessation programs in the world include the contents of the app, professional mentoring, and pharmacotherapy. Bricker et al [20] evaluated a smoking cessation app delivering ACT in 99 adult smokers and found that 11% had achieved 30-day point prevalence abstinence in an intent-to-treat (ITT) analysis at the 2-month follow-up. The app assessed by Iacoviello et al [16] has a series of missions and personalized messages that adhere to the United States Clinical Practice Guideline, and 26.2% of the ITT sample (109/416) reported 30-day abstinence from smoking after 8 weeks. Another study evaluated a comprehensive, multiphase digital smoking cessation program that includes a mobile carbon monoxide breath sensor and text-based human coaching and reported a 30-day point prevalence abstinence of 27.6% (88/319, ITT) [21]. The outcomes could have been strongly affected by the difference in the country the study was conducted in and ethnicity. Also, the study design, program duration, baseline characteristics, and outcome measurement are not consistent among these programs, including the ascure program. We acknowledge that the huge differences limit the comparability of these programs, but the efficacy of the ascure program is favorable when assessing 1-month smoking cessation success.

Second, proactive use of the smartphone app (eg, keeping a digital diary and watching educational videos) during the first 12 weeks of the program was significantly associated with CARs during weeks 21-24. Keeping a smoking cessation diary is recommended by various Japanese academic societies (eg, the Cardiovascular Society, Lung Cancer Society, Cancer Society, and Respiratory Society) [22]. This study further supports the concept that writing diaries is associated with an increased success rate in smoking cessation. The number of diary entries might reflect an individual’s willingness to quit smoking and therefore their commitment to the program. A study comparing two other smartphone-based smoking cessation interventions — SmartQuit and Quit guide — reported that the number of times the application was opened was a significant predictor of smoking cessation [23]. In addition, another study on the app-based program, Clickotine, also found that the number of weeks with more than 1 interaction in the app was associated with smoking cessation success [16]. Participant engagement has been found to be a consistent predictor of smoking cessation in several programs, and interactivity might be a key factor in improving engagement [15,16,23-26]. The proactive use of a smartphone app as an indicator of patient motivation might be a predictive factor of better outcomes of smoking cessation programs.

Third, the ascure program seemed to attract more relatively young, female participants with low dependence, as represented by the Brinkman index. This may imply that, because of the completely remote aspect of the ascure program, it could recruit people who do not utilize the outpatient service voluntarily.

### Strengths and Limitations

This study was the first to examine the efficacy of a novel smoking cessation program for members of a Japanese health insurance society. We used a biochemically validated smoking status test to assess the efficacy of the program. However, this study has several limitations. First, the absence of a control group is a significant problem when assessing the efficacy of the program. Our finding that a predictor of smoking cessation was the intensive use of the program could merely reflect that motivated patients made more intensive use of the program. Future studies, such as crossover trials with a control group, are necessary to better evaluate the efficacy of the program. Second, several factors could limit the generalizability of the findings: participants’ level of motivation to engage in the program, participants’ sociodemographic status (all were members of corporate health insurance societies), and access to technology (ie, smartphones). Third, we evaluated 1-month continuous abstinence (as does the outpatient service in Japan). This limits the comparability with internet-based programs in other countries. Fourth, data on adherence to nicotine patches and the quality of video counseling, which might affect outcomes, were not collected. Finally, it may be necessary to evaluate smoking status even further into the future, such as at week 52 or beyond, to confirm the long-term efficacy of this program.

### Conclusion

The completely remote ascure smoking cessation program achieved favorable smoking cessation success rates while improving accessibility and adherence to a cessation program. Moreover, proactive use of the smartphone app may have contributed to successful smoking cessation over a relatively long period. The combination of internet-based counseling, a smartphone app, and over-the-counter medications might be a viable solution for long-term smoking cessation.

## Appendix

Multimedia Appendix 1 Interface of the ascure smoking cessation app.

## Abbreviations

ACT: acceptance and commitment therapy
CAR: continuous abstinence rate
FTND: Fagerström Test for Nicotine Dependence
IQR: interquartile range
ITT: intention-to-treat
KTSND: Kano Test for Social Nicotine Dependence
TDS: Tobacco Dependence Screener
